# Efficacy and safety of a combination of azithromycin and chloroquine for the treatment of uncomplicated *Plasmodium falciparum* malaria in two multi-country randomised clinical trials in African adults

**DOI:** 10.1186/1475-2875-13-458

**Published:** 2014-11-25

**Authors:** Issaka Sagara, Abraham R Oduro, Modest Mulenga, Yemou Dieng, Bernhards Ogutu, Alfred B Tiono, Peter Mugyenyi, Ali Sie, Monique Wasunna, Kevin C Kain, Abdoulaye A Djimdé, Shirsendu Sarkar, Richa Chandra, Jeffery Robbins, Michael W Dunne

**Affiliations:** Malaria Research and Training Centre, University of Sciences, Techniques and Technologies, Bamako, Mali; Navrongo Health Research Centre, Navrongo, Ghana; Tropical Diseases Research Centre, Ndola, Zambia; Parasitology and Mycology Department, Faculty of Medicine, Pharmacy, and Odonto-Stomatology, University of Dakar, Dakar, Senegal; Walter Reed Project, Centre for Clinical Research, Kenya Medical Research Institute, Kisumu, Kenya; Centre National de Recherche et de Formation sur le Paludisme, Ouagadougou, Burkina Faso; Joint Clinical Research Centre, Kampala, Uganda; Centre de Recherche en Sante de Nouna, Nouna, Burkina Faso; Kenya Medical Research Institute, Nairobi, Kenya; Sandra A Rotman Laboratories, Sandra Rotman Center for Global Health, University Health Network, Toronto General Hospital, University of Toronto, Toronto, Ontario Canada; Sciformix, Mumbai, India; Formerly of Pfizer Inc, New York, NY USA; Global Research and Development, Pfizer Inc, 445 Eastern Point Road, Groton, CT 06340 USA; Durata Therapeutics, Inc, Morristown, NJ USA

**Keywords:** Azithromycin, Chloroquine, Falciparum malaria, Combination treatment

## Abstract

**Background:**

Given increasing rates of resistance to existing therapy, new options for treatment and prophylaxis of malaria are needed.

**Methods:**

Two randomised, comparative, non-inferiority studies were conducted in Africa, one double-blinded and one open-label. Adults with fever, a positive peripheral blood smear, and a positive rapid diagnostic test for *Plasmodium falciparum* were randomised in both studies to either azithromycin (AZ) 1,000 mg plus chloroquine (CQ) 600-mg base (AZCQ 1,000 mg) once daily for three days or mefloquine hydrochloride (MQ) 1,250 mg (split dose). In the first study, an additional regimen of AZ 500 mg plus CQ 600-mg base (AZCQ 500 mg) once daily for three days was included. All study participants were hospitalised until three consecutive daily blood smears were negative for asexual *P. falciparum* parasitaemia. Study participants were evaluated weekly for 42 days, with Day 28 polymerase chain reaction (PCR)-corrected parasitological clearance rate as primary endpoint.

**Results:**

A total of 467 subjects were randomised in the two studies. At 28 days’ follow-up, PCR-corrected parasitological clearance rates in the per protocol population in the first study were 101/103 (98%) with AZCQ 1,000 mg compared with 102/103 (99%) with MQ (95% confidence interval [CI]: -5.2, 3.3). The AZCQ 500-mg regimen was stopped during an interim study review (six [86%] clearance of seven evaluable; two lost to follow-up). In the second study, clearance rates were similar: AZCQ 1,000 mg 107/107 (100%) *vs* MQ 111/112 (99%; 95% CI: -1.8, 3.6). Among the participating countries, *in vitro* CQ resistance based on *pfcrt* mutation frequency in the baseline isolates across both studies ranged from 20.8% (Zambia) to 96.1% (Uganda). Serious adverse events (AEs; all causality) were observed more frequently with MQ compared with AZCQ (four *vs* one, respectively), though discontinuations for AEs were similar (four *vs* three, respectively). Common AEs in the AZ-containing arms included pruritus, vomiting, dizziness, and headache.

**Conclusions:**

Among adults with symptomatic uncomplicated falciparum malaria in Africa, the combination of AZ 1,000 mg and CQ 600-mg base once daily for three days resulted in Day 28 PCR-corrected parasitological clearance rates of ≥98% and was non-inferior to treatment with MQ. AZCQ was well tolerated.

**Trial registration:**

ClinicalTrials.gov identifiers NCT00082576 and NCT00367653

**Electronic supplementary material:**

The online version of this article (doi:10.1186/1475-2875-13-458) contains supplementary material, which is available to authorized users.

## Background

Malaria remains one of the most common causes of morbidity and mortality in Africa. In 2011, >80% of the population in most countries in Africa were at high risk for malaria [1]. The use of pesticides and the broad availability of chloroquine (CQ) effectively contained malaria on the African continent through the 1970s, until subsequent development of resistance by the *Plasmodium* species led to increased morbidity and mortality [2].

Management of malaria in Africa requires a variety of preventive and treatment initiatives, each of which targets different patient subpopulations. As a consequence, these initiatives demand medical therapy that, individually, vary in the requirements for safety, efficacy, and tolerability and, collectively, must integrate within a larger public health strategy. Increasing degrees of parasite resistance to mainstream drug interventions have further limited the choice of therapy available for malaria [3]. For example, there is currently no generally acceptable alternative for sulphadoxine/pyrimethamine in the intermittent preventive therapy in pregnancy programme. New therapy is needed and alternative options must be explored for their potential utility.

The anti-malarial properties of azithromycin (AZ) have been documented *in vitro* and in animal experiments, as well as in treatment and prevention clinical trials of both *Plasmodium falciparum* and *Plasmodium vivax*[4–8]. Given as monotherapy, AZ did not meet clinical standards of efficacy for treatment of falciparum malaria. However, further *in vitro* work suggested the possibility of synergy between AZ and CQ [4], which was subsequently supported by a small clinical trial in India [6].

Given the need for a new anti-malarial therapy and the scarcity of new potential agents, and the promising early results of a pilot clinical study [6], as well as the well-established safety profiles of both agents, there was a need to conduct a study to compare combined AZ and CQ with mefloquine hydrochloride (MQ). MQ was used as the comparator based on the recommendation of the US Food and Drug Administration (FDA). The results of two trials of the combination of AZ and CQ in the treatment of symptomatic uncomplicated *P. falciparum* malaria in adults in Africa are reported here.

## Methods

### Study design

The two phase II/III multi-centre, parallel, randomised, controlled, comparative, non-inferiority studies reported here were performed sequentially. Study number A0661134 (referred to hereafter as study 1134; ClinicalTrials.gov identifier NCT00082576; double-blind phase II/III) was conducted between June 2004 and May 2006, and study number A0661155 (referred to hereafter as study 1155; ClinicalTrials.gov identifier NCT00367653; open-label phase III) was conducted between November 2006 and September 2007. Both studies were conducted in African countries; study 1134 in Ghana, Mali, Zambia, Kenya, and Uganda and study 1155 in Ghana, Mali, Zambia, Kenya, Burkina Faso, and Senegal. In both trials, the comparator agent was MQ and both studies were identical in conduct and analysis with two exceptions. Study 1134 was conducted with blinded therapy and initially included a third arm (AZ 500 mg plus a CQ 600-mg base [AZCQ 500 mg] once daily for three days), whereas in study 1155 medication was provided unblinded with the same two treatment regimens as in study 1134. As a consequence, the methods described below relate to study conduct and analysis for both trials with exceptions specifically noted where relevant.

### Study population

Eligible subjects were ≥18 years of age with symptomatic uncomplicated malaria as demonstrated by blood smears positive for *P. falciparum* asexual parasitaemia between 1,000 and 100,000 parasites/μL and fever, documented or by history, within the prior 24 hours. Subjects also were required to have a serum glucose ≥60 mg/dL and a rapid diagnostic test positive for *P. falciparum*. Women of childbearing potential were required to have a negative urine human chorionic gonadotropin test before study entry, and enrolled subjects were to use adequate contraception during the entire study. Subjects were excluded for any of the following reasons: clinical or laboratory evidence of severe or complicated malaria; the presence of non-falciparum species on microscopy; pregnancy or breast-feeding; history of allergy or hypersensitivity to AZ, CQ, MQ, or related compounds; history of epilepsy or psoriasis; treatment with any anti-malarial drug or with any antibacterial with known anti-malarial activity within two weeks before enrolment into the study; laboratory evidence of abnormal renal or liver function; any major psychiatric disorders; inability to swallow oral medication; treatment with other investigational drugs 30 days prior to enrolment; alcohol and/or any other drug abuse; requirement to use medication during the study that might have interfered with the evaluation of the study drug; other medical conditions that would have interfered with the evaluation of the therapeutic response or safety of the study drug; and inability to comprehend and/or unwillingness to follow the study protocol; or, prior participation in this study. Finally, subjects were excluded if they had not lived continuously in a malaria-endemic area for at least the previous year.

Subjects were to be withdrawn from the study and placed on alternative therapy for any of the following reasons: impaired consciousness, respiratory distress, seizures, hypoglycaemia, gross haematuria, increase in parasitaemia to >100,000 parasites/μL 48 hours after the first treatment dose, failure of parasitaemia to decrease to 25% of the baseline value on Day 2 and investigator opinion that other anti-malarial therapy was indicated or treatment failure.

### Drug treatment

Study drug was administered as blinded therapy with matching placebo (in study 1134) or unblinded (in study 1155). In study 1134, eligible subjects were initially randomised to oral treatment for three days in one of three active treatment arms: AZ 1,000 mg plus a CQ 600-mg base (AZCQ 1,000 mg) once daily for three days, AZ 500 mg plus a CQ 600-mg base (AZCQ 500 mg) once daily for three days, or MQ (salt equivalent to 28-mg free base) 1,250 mg (initial 750-mg dose followed by 500 mg on Day 0). In study 1155, subjects were randomised to either AZCQ 1,000 mg once daily for three days or MQ (salt equivalent to 28-mg free base) 1,250 mg (initial 750-mg dose followed by 500 mg on Day 0). MQ was used as the comparator based on the recommendation of the FDA, as this was the only FDA-approved oral anti-malaria agent at the time. MQ was expected to have high efficacy rates in all of the chosen study locations at the time of the study conduct.

### Parasite identification

A blood dipstick-based test (Binax NOW® ICT, Scarborough, ME, USA) was used for rapid diagnostic testing of *P. falciparum*. Giemsa-stained blood smears were read and interpreted by experienced microscopists who were blinded to all clinical information, including treatment allocation and the readings by the other microscopists. Three slides were prepared for each subject; two of these were read locally, whereas the third slide was maintained for possible third party review in case of any discordance ≥50% between the first two readers. Microscopy results at the site guided subject management; any subject with persistent or recurrent parasitaemia during the follow-up period was treated with anti-malarial drugs according to local treatment guidelines and withdrawn from the study after documentation of clearance of parasitaemia.

For subjects who developed asexual parasitaemia after a period of clearance, paired blood blots from baseline and the time of recurrence were analysed to distinguish recrudescence from re-infection. Genotyping was performed by nested polymerase chain reaction (PCR) at two different laboratories, the Naval Medical Research Unit 2 in Jakarta, Indonesia (study 1134 only) and the Malaria Research and Training Centre in Bamako, Mali (both studies). In both studies, merozoite surface protein (MSP)-1, MSP-2, and the microsatellite CA1 gene loci (Su) were amplified at the Malaria Research and Training Centre. Recrudescence was defined as the reappearance of asexual blood-stage parasites of the same genotype as Day 0 parasites, whereas re-infection was defined as infection by a different genotype. Subjects were censored at the time of re-infection.

Paired blood specimens, collected before treatment and at the time of recurrent asexual parasitaemia (treatment failure), were evaluated for mutations in the *P. falciparum* CQ resistance transporter gene (*pfcrt*). For study 1134, molecular detection of the K76T mutation associated with CQ resistance was detected by means of a standardised real-time PCR-based diagnostic assay [9, 10]. In addition, specimens collected on Day 0 from subjects who responded to treatment also were analysed for *pfcrt* gene mutations. Based on differential melting-curve analysis, isolates were identified as CQ-sensitive isolates with a CVMNK haplotype or CQ-resistant isolates carrying a CVIET, SVMNT, CVMNT, or CVMET haplotype. For study 1155, nested PCR methods were performed for *pfcrt* (K76T) and *P. falciparum* multi-drug resistance protein-1 (*pfmdr1*; N86Y) with the specific primer pairs followed by restriction fragment length polymorphism [11].

### Study assessments

All subjects were hospitalised and monitored until three consecutive blood smears were negative for asexual *P. falciparum* parasitaemia and the investigator deemed discharge from the hospital appropriate. Peripheral blood smears for parasite counts were obtained at eight-hour intervals until clearance was demonstrated, then on Day 7 and weekly thereafter through Day 42 to monitor recrudescence. Vital signs, clinical signs and symptoms, adverse events (AEs), and concomitant medications were assessed for all subjects on each day of treatment (Days 0, 1, and 2) and at each post-therapy visit (Days 7, 14, 21, 28, 35, and 42). Haematology and serum chemistry laboratory tests were performed at Baseline and Day 3 and at subsequent visits if clinically indicated. Haematology tests included red blood count, white blood count with differential, haemoglobin, haematocrit, and platelets. Serum chemistry tests included electrolytes, urea nitrogen, creatinine, aspartate aminotransferase, alanine aminotransferase, and alkaline phosphatase.

### Efficacy

The primary endpoint was the asexual *P. falciparum* parasite clearance rate, adjusted for molecular testing (PCR-corrected) to differentiate recrudescence (true failures) from re-infection. Efficacy was assessed at Days 28 (primary endpoint) and 42 in the per protocol (PP) population. Treated subjects were excluded from the PP population if they did not meet the disease definition, did not receive all three days of study medication (unless designated a treatment failure, defined as the development of signs of severe malaria in the presence of parasitaemia), or did not have a blood smear at the specified time point (unless due to recurrent parasitaemia before that time point). Subjects were assigned a response of eradicated if parasitaemia cleared within seven days after initiation of treatment and did not recur through the time point of interest. Failure was defined as not achieving clearance of asexual *P. falciparum* parasitaemia within seven days or, if after achieving clearance, PCR-corrected parasitaemia recurred.

Secondary efficacy analyses included time required for asexual parasite clearance, fever clearance time, and assessment of the percentage of subjects with early and late treatment failures as defined by the World Health Organization [12].

### Safety

All subjects who received at least one dose of study medication were evaluated for safety. Subjects were monitored closely for clinical evidence of illness progression. All subjects with persistent or recurrent parasitaemia received therapy consistent with the local standard of care and were monitored until parasite clearance was documented. Other safety evaluations included AE and vital sign monitoring throughout the study, haematology and serum chemistry laboratory evaluations, and physical examinations.

### Statistical analyses

The primary efficacy analysis compared the PCR-corrected asexual *P. falciparum* parasite clearance rates in the AZCQ 1,000 mg and MQ treatment arms at Day 28 in the PP population. A two-sided confidence interval (CI), using the appropriate confidence level, was constructed for the difference between treatment groups in asexual parasite clearance rates using normal approximation to the binomial, and also for within group rates. A CI was computed for the clearance rate within a treatment group using exact methods when no failures were observed. The confidence level in the final analysis for study 1134 required a small adjustment upward from 95 to 95.04% to prevent the overall false-positive rate from exceeding 0.05, due to the planned interim analysis of the primary efficacy outcome. There was no adjustment for centres in the analyses. Non-inferiority was to be concluded if the lower boundary of the CI for the difference in parasite clearance rates (AZCQ 1,000 mg – MQ) was -10% or more. A non-inferiority margin of 10% was based on regulatory guidance available at the time of study conception. Clearance time comparisons up to Day 7 were generated using Kaplan-Meier estimates. Parasite clearance time was defined as the time in days from Baseline to the first of the three consecutive zero parasite counts. Fever clearance time was defined as the time in days from Baseline to the first of the two consecutive time points without evidence of elevated temperature.

Total sample size was determined based on the following assumptions: 80% power to show non-inferiority of the AZCQ 1,000-mg arm relative to the MQ arm, 85% of randomised subjects satisfying criteria for inclusion in the PP analysis at the Day 28 visit, and expected parasite clearance rates of 95% in the AZCQ 1,000-mg arm and 95% in the MQ arm.

These studies were conducted in compliance with the Declaration of Helsinki, institutional review boards, informed consent regulation, and the International Conference on Harmonisation Good Clinical Practice guidelines. All local regulatory requirements were followed. The clinical protocol was conducted in accordance with FDA regulations. Pfizer Inc conducted the clinical monitoring of the study.

## Results

**Figure 1:**
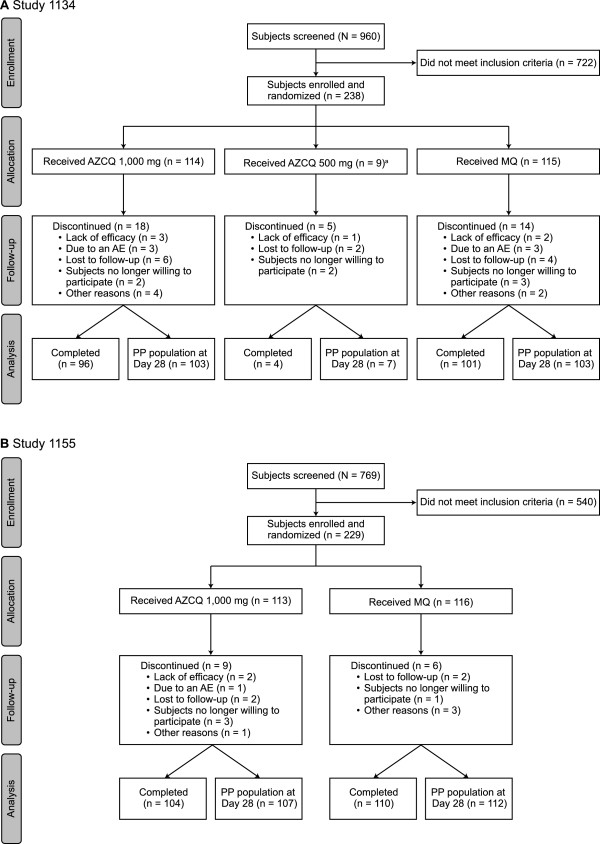
**Subject disposition in study 1134 (A) and study 1155 (B).** ^a^The AZCQ 500-mg regimen was not included in the analysis, as treatment was stopped early on the recommendation of the data safety monitoring board after a review of data from studies conducted in South America and India demonstrated a dose response with lower efficacy rates for this regimen compared with AZCQ 1,000 mg. AE, adverse event; AZCQ 1,000 mg, azithromycin 1,000 mg plus chloroquine 600-mg base; AZCQ 500 mg, azithromycin 500 mg plus chloroquine 600-mg base; MQ, mefloquine hydrochloride; PP, per protocol.

A total of 467 subjects were randomised to study medication (Figure 1). The demographic and baseline characteristics of subjects were similar among treatment allocations (Table 1). Similar numbers of subjects on each regimen completed therapy.

The AZCQ 500-mg regimen was stopped early in study 1134 on the recommendation of the data safety monitoring board after a review of data from studies conducted in South America and India demonstrated a dose response with lower efficacy rates for this regimen compared with AZCQ 1,000 mg. At that point, a total of nine subjects had been randomised to the AZCQ 500-mg regimen, of which six of seven subjects in the PP population had responded to therapy through Day 28 (Figure 1).

**Table 1:** **Subject demographics**

	Study 1134		Study 1155	
	AZCQ 1,000 mg (N = 114)	MQ 1,250 mg (N = 115)	AZCQ 1,000 mg (N = 113)	MQ 1,250 mg (N = 116)
Sex, n				
Male	66	61	65	63
Female	48	54	48	53
Age group, years, n				
18-44	106	101	100^a^	99
45-64	8	13	13	15
≥65	0	1	0	2
Mean (SD)	29.4 (9.6)	30.2 (11.0)	30.2 (11.0)	31.2 (12.4)
Weight, kg				
Mean (SD)	61.4 (10.8)	60.4 (11.0)	60.0 (9.9)	59.4 (10.1)
Range	40.0-105.0	39.0-107.0	40.0-83.6	39.0-94.0
Baseline parasite count/μL, mean ± SD	20,889 ± 24,176	20,146 ± 27,564	16,686 ± 22,863	17,651 ± 22,435
Range	160-111,040	1,040-164,600	120-93,440	1,000-97,234
Number of episodes of malaria in previous 2 years				
0	61	66	69	67
1	20	18	9	14
2	15	9	16	18
≥3	17	22	19	17

^a^One subject <18 years of age.

AZCQ 1,000 mg, azithromycin 1,000 mg plus chloroquine 600-mg base; MQ, mefloquine hydrochloride; SD, standard deviation.

All subjects with persistent or recurrent parasitaemia during the follow-up period were to receive rescue therapy with an anti-malarial regimen that was consistent with the local standard of care. Ten and five subjects treated with AZCQ and five and three patients treated with MQ in studies 1134 and 1155, respectively, received rescue medication. The most common rescue mediation used was quinine in both treatment groups.

Among study 1134 PP population subjects at Day 28, those randomised to AZCQ 1,000 mg had 98.1% parasite clearance (94.9, 100 95.04% CI; n = 101 of 103) compared with 99.0% parasite clearance (96.7, 100 95.04% CI; n = 102 of 103) in those who received MQ based on PCR-corrected results (Table 2; difference, -0.97% [95.04% CI: -5.2, 3.3]). For corresponding PCR-uncorrected results, those randomised to AZCQ 1,000 mg had 95.1% parasite clearance (90.5, 99.8 95.04% CI; n = 98 of 103) compared with 98.1% parasite clearance (94.9, 100 95.04% CI; n = 101 of 103) in those who received MQ. At Day 42, 99% parasite clearance was observed in both groups (PCR-corrected; 96.6, 100 95.04% CI; n = 100 of 101 AZCQ; 96.6, 100 95.04% CI; n = 100 of 101 MQ; difference, 0% [95.04% CI: -3.74, 3.74]).

**Table 2:** **Summary of efficacy outcomes at Day 28 in the parasitological per protocol population**

	Study 1134		Study 1155	
	AZCQ 1,000 mg	MQ 1,250 mg	AZCQ 1,000 mg	MQ 1,250 mg
All treated, N	114	115	113	116
PP population, n	103	103	107	112
PCR-corrected				
Eradicated, n (%) (95% CI)	101 (98.1) (94.9, 100)^a^	102 (99.0) (96.7, 100)^a^	107 (100.0) (96.6, 100)	111 (99.1) (96.9, 100)
Difference, % (95% CI)	-0.97 (-5.23, 3.29)^a^		0.89 (-1.77, 3.56)	
ETF, n (%)	0	1 (1.0)	0	1 (0.9)
LTF, n (%)	2 (1.9)	0	0	0
LPF, n (%)	2	0	0	0
LCF, n (%)	0	0	0	0
Median fever clearance time, days	<0.5	0.5	1.5	1.0
Median parasite clearance time, h	48	36	44	40

^a^95.04% CI (small adjustment to the 95% CI required in order to account for a planned interim look at the primary efficacy outcome).

AZCQ 1,000 mg, azithromycin 1,000 mg plus chloroquine 600-mg base; CI, confidence interval; ETF, early treatment failure; LCF, late clinical failure; LPF, late parasitological failure; LTF, late treatment failure; MQ, mefloquine hydrochloride; PCR, polymerase chain reaction; PP, per protocol.

The rate of gametocyte clearance was similar in both AZCQ and MQ groups in both studies and was >95% from Day 7 onwards.

The median time to clearance of asexual *P. falciparum* parasitaemia was 48 hours for those given AZCQ 1,000 mg and 36 hours for those given MQ (p = 0.0226). Median time to resolution of fever was observed to be <0.5 days after starting therapy for subjects randomised to AZCQ 1,000 mg and 0.5 days for those given MQ.

Similar findings were observed in study 1155, whereas subjects in the PP population randomised to AZCQ 1,000 mg had 100% parasite clearance (96.6, 100 95% CI; n = 107 of 107) compared with 99.1% (96.9, 100 95% CI; n = 111 of 112) in those randomised to MQ (difference, 0.89% [95% CI: -1.8, 3.6]) at Day 28. For corresponding PCR-uncorrected results, subjects in both the AZCQ 1,000-mg (96.8, 100 95% CI; n = 106 of 107) and MQ (96.9, 100 95% CI; n = 111 of 112) groups had a 99.1% parasite clearance. At Day 42, PCR-corrected parasite clearance remained at 100% for the AZCQ group (96.5, 100 95% CI; n = 104 of 104) and 99.1% for the MQ group (96.9, 100 95% CI; n = 110 of 111; difference, 0.90% [95% CI: -1.8, 3.6]).

The median time to clearance of parasitaemia was 44 hours for subjects given AZCQ 1,000 mg and 40 hours for those given MQ. Median time to resolution of fever was observed to be 1.5 days after starting therapy for subjects randomised to AZCQ 1,000 mg and 1 day for those given MQ, although the difference was not statistically significant.

Two late parasitological failures (LPFs) were observed in study 1134 for subjects in the AZCQ 1,000-mg treatment group (Table 2), whereas there were no LPFs in the MQ treatment group. No LPFs were observed in study 1155 in either treatment group.

Paired isolates from the two subjects who demonstrated a recrudescence on AZCQ 1,000 mg were sequenced to identify possible signature resistance mutations. No such mutations were identified [13]. CQ–resistance-associated mutation rates varied among the countries participating in these studies, from 96.1% in Uganda (study 1134) to 20.8% in Zambia (study 1155; Table 3). An analysis of isolates collected during study 1134 indicated that 13% (15/115) of CQ-resistant isolates carried the CVMNT *pfcrt* haplotype (Table 4).

**Table 3:** **Markers of chloroquine resistance in isolates obtained during the clinical trial**

Number of subjects		***Pfcrt***			Resistance mutations, %		
	Tested	K	T	KT	No band	2004-2006 ^a^	2006-2007 ^b^
Ghana							
Study 1134	27	11	16	0	0	59.3	
Study 1155	83	52	25	3	3		33.7
Uganda							
Study 1134	51	1	49	0	1	96.1	
Study 1155							
Zambia							
Study 1134	116	85	26	5	0	26.7	
Study 1155	77	61	13	3	0		20.8
Mali							
Study 1134	27	8	18	0	1	66.7	
Study 1155	29	5	20	4	0		82.8
Burkina Faso							
Study 1134							
Study 1155	27	19	7	0	1		25.9
Kenya							
Study 1134	15	2	10	0	3	66.7	
Study 1155	7	1	5	0	1		71.4
Senegal							
Study 1134							
Study 1155	6	3	2	1	0		50.0

^a^Study 1134.

^b^Study 1155.

K, wild type; KT, mixed; *pfcrt*, *Plasmodium falciparum* chloroquine resistance transporter gene; T, resistant.

**Table 4:** **Number of subjects with** ***pfcrt*** **gene mutations in study 1134**

	AZCQ 1,000 mg (N = 114)	MQ 1,250 mg (N = 114)
CQ-sensitive, n	48	59
CVMNK	48	58
SVMNK	0	1
CQ-resistant, n	63	52
CVMET	0	1
CVMNT	9	6
CVIET	54	45
Negative, n	3	2
Other, n	0	1

AZCQ 1,000 mg, azithromycin 1,000 mg plus chloroquine 600-mg base; CQ, chloroquine; MQ, mefloquine hydrochloride; *pfcrt*, *Plasmodium falciparum* CQ resistance transporter gene.

Treatment-emergent AEs considered related to study medication were observed in 78.1% (89/114) of subjects randomised to AZCQ 1,000 mg and in 61.7% (71/115) of those randomised to MQ in study 1134, and in 70.8% (80/113) and 62.1% (72/116) of subjects, respectively, in study 1155 (Table 5). Pruritus was seen more frequently with AZCQ 1,000 mg, whereas dizziness was seen more often with MQ (Table 5). Overall, three (1.3%) subjects given AZCQ 1,000 mg discontinued therapy due to an AE related to study drug (vomiting, pruritus, vomiting/dizziness/tinnitus) compared with two (0.9%) of those receiving MQ (fever/hypotension/haematuria, hypertension/vomiting). No clinically significant changes were observed in any laboratory values over the duration of the study and abnormal laboratory values did not result in any study discontinuations (see Additional files 1 and 2).

**Table 5:** **Treatment-emergent, treatment-related adverse events in ≥5% of subjects in any group in each study**

	Study 1134		Study 1155	
	AZCQ 1,000 mg (N = 114)	MQ 1,250 mg (N = 115)	AZCQ 1,000 mg (N = 113)	MQ 1,250 mg (N = 116)
Any AE, n (%)	89 (78.1)	71 (61.7)	80 (70.8)	72 (62.1)
Pruritus	58 (50.9)	11 (9.6)	32 (28.3)	1 (0.9)
Dizziness	11 (9.6)	26 (22.6)	18 (15.9)	19 (16.4)
Vomiting	18 (15.8)	12 (10.4)	4 (3.5)	20 (17.2)
Headache	15 (13.2)	11 (9.6)	20 (17.7)	25 (21.6)
Abdominal pain	8 (7.0)	13 (11.3)	13 (11.5)	9 (7.8)
Nausea	9 (7.9)	13 (11.3)	10 (8.8)	12 (10.3)
Asthenia	6 (5.3)	11 (9.6)	9 (8.0)	3 (2.6)
Palpitations	3 (2.6)	7 (6.1)	–	–
Diarrhoea	6 (5.3)	5 (4.3)	11 (9.7)	4 (3.4)
Fatigue	–	–	4 (3.5)	6 (5.2)
Pain	2 (1.8)	2 (1.7)	6 (5.3)	1 (0.9)

AE, adverse event; AZCQ 1,000 mg, azithromycin 1,000 mg plus chloroquine 600-mg base; MQ, mefloquine hydrochloride.

Overall in both studies, treatment-related serious AEs were only observed in the MQ group (3/231 [1.3%]; mental disorder, nephrotic syndrome/blood creatinine increased and intentional self-injury). A severe AE of vomiting was seen in one (0.4%) subjects given AZCQ 1,000 mg, whereas six severe AEs were observed in five (2.2%) subjects given MQ (vomiting, increased creatinine, psychosis, intentional self-injury, nephrosis, and dizziness). The majority of all other AEs with AZCQ 1,000 mg and MQ were mild in severity.

## Discussion

The fixed-dose combination of AZ 1,000 mg and a CQ 600-mg base was found to be non-inferior to MQ in two randomised studies conducted in Africa in adults with symptomatic uncomplicated malaria due to *P. falciparum*. In addition to similar clinical and parasitological outcomes, the AZCQ fixed-dose combination also achieved an overall efficacy rate of 99%, which is consistent with other treatment modalities recommended in treatment guidelines for this subject population [12]. The mean time to resolution of parasitaemia was comparable in each regimen, as was the time to resolution of fever.

A pre-specified non-inferiority margin of 10% was based on regulatory guidance at the time of study conception. Although this margin has been used by other phase III trials as recent as 2011, many trials of late have switched to a more conservative margin of 5% [14]. Given the level of parasite clearance achieved at Day 28 in studies 1134 and 1155, lower limits of the CIs comparing the two treatments would indicate a difference relative to MQ of no worse than 5.23% and 1.77%, respectively.

In Zambia, the rate of clinical resistance to CQ was >70% in 2003 at the time of switching from CQ to artemether/lumefantrine. Based on this observation, it is clear that AZCQ displayed efficacy in areas with a high prevalence of CQ-resistant falciparum malaria.

Molecular marker resistance to CQ across these seven African countries between 2004 and 2007 ranged from approximately 21 to 96%. There was a trend to lower rates of CQ resistance in the clinical trial sites in Ghana and Zambia over this time period, which may be related to anti-malarial policies in these countries *vs* the other countries included in this study.

The *pfcrt* CQ-resistant haplotype most frequently described in Africa has been CVIET, which is thought to have possibly been introduced from southeast Asia through India [10]. As previously reported, the CVIET haplotype was again observed in this study. Notably, 13% of isolates in this study carried the CVMNT haplotype and were recovered from Ghana and Zambia. Both the SVMNT and CVMNT CQ-resistant mutations have been previously observed in Africa [15, 16].

While no CQ monotherapy control arm was included in this trial, the historical rates of clinical response to CQ in areas with significant *in vitro* CQ resistance would be expected to be significantly lower than those observed on this combination therapy [17]. Whether the enhanced efficacy of the combination is a consequence of the direct effects of AZ on the parasite or the effect of AZ on the resistance mechanisms mediating CQ resistance, or both, cannot be established from these data.

Moreover, anti-malarial immunity in malaria-endemic areas also can assist in parasite clearance and may play a role in the enhanced efficacy of the combination therapy, although innate immunity is equally likely to be effective in those subjects who received either AZ or CQ alone [18].

Discontinuations due to AEs were infrequent and numbers were similar in each regimen. Pruritus was seen more frequently with the AZCQ combination, a well-described effect associated with the use of CQ in subjects with melanoderma [19]. Central nervous system AEs were seen more frequently with MQ, and again this is consistent with historical experience [20]. Gastrointestinal AEs occurred with both regimens to a similar degree.

For a regimen to be considered appropriate as a therapy for malaria, it first must demonstrate that it is safe and effective in the target subject population. Other considerations, however, should be addressed with regard to the AZCQ combination. CQ is no longer recommended for the treatment of malaria in Africa due to the generally high rates of background CQ resistance and subsequent poor clinical response to therapy throughout the continent. The position of the World Health Organization is that all therapy for the treatment of malaria should be combination therapy, primarily combinations that include an artemisinin derivative [21]. The combination therapy should include a partner drug with sufficient efficacy to protect the artemisinin from the development of resistance. Alternatively, new modelling suggests that the introduction of a variety of therapy may be more effective in managing the rate at which resistance appears [22].

A limitation of this study was the recruitment of adults with symptomatic uncomplicated malaria only and thus, other target populations, including pregnant women, should be studied, as it is known that pregnant women are more susceptible to malaria compared with non-pregnant women within the same age group [23]. The progressive decline in the efficacy of sulphadoxine/pyrimethamine in Africa, concerns with artemisinin use in pregnancy [12], and the extensive safety database for both AZ and CQ suggest that studies to investigate the merits of this combination for the prevention of malaria during pregnancy would appear justified [24]. Other areas of study also may include use in the paediatric population and, given the activity of AZ in a variety of bacterial infections [25, 26], home-based management of fever.

A phase III trial (ClinicalTrials.gov identifier NCT01103063) that evaluated a blended tablet of AZ and CQ for intermittent preventive treatment of malaria in pregnancy was stopped early, as an interim analysis indicated it was unlikely to meet the primary endpoint (i.e., stopped for trial futility) defined as superiority of AZCQ over sulfadoxine/pyrimethamine in the proportion of patients meeting a sub-optimal pregnancy outcome (abortion [≤28 weeks], stillbirth [>28 weeks], premature delivery [<37 weeks], low birth weight [<2,500 g] live neonate, missing neonatal birth weight, or lost to follow-up). As the primary endpoint was analysed using the intent-to-treat population, lack of tolerability of the combination treatment may have resulted in a higher dropout rate leading to the inclusion of missing data as a failure in the interim analysis.

New therapy for the prevention and treatment of malaria in the context of a broader campaign of public health interventions is, therefore, needed to help limit the morbidity and mortality associated with this disease.

## Conclusions

The results of this study indicate that among adults with symptomatic uncomplicated falciparum malaria in Africa, a fixed-dose combination of AZ 1,000 mg and a CQ 600-mg base once daily for three days resulted in Day 28 and Day 42 PCR-corrected parasitological clearance rates of ≥98%. AZCQ was non-inferior to treatment with MQ and was well tolerated.

## Electronic supplementary material

Additional file 1: Table S1: Median change from Baseline to last observation in laboratory values in study 1134. Description: The data in the table provides details of laboratory values from study 1134. (DOCX 38 KB)

Additional file 2: Table S2: Median change from Baseline to last observation in laboratory values in study 1155. Description: The data in the table provides details of laboratory values from study 1155. (DOCX 35 KB)

## Abbreviations

AE: Adverse event
AZ: Azithromycin
AZCQ: Azithromycin chloroquine combination
AZCQ 500 mg: Azithromycin 500 mg plus chloroquine 600-mg base
AZCQ 1: 000 mg: Azithromycin 1,000 mg plus chloroquine 600-mg base
CI: Confidence interval
CQ: Chloroquine
ETF: Early treatment failure
FDA: US Food and drug administration
LCF: Late clinical failure
LPF: Late parasitological failure
MQ: Mefloquine hydrochloride
MSP: Merozoite surface protein
PCR: Polymerase chain reaction
LTF: Late treatment failure
*pfcrt*: *Plasmodium falciparum* CQ resistance transporter gene
PP: Per protocol
SD: Standard deviation.
