# Supplementary material for: Efficacy and safety of a combination of azithromycin and chloroquine for the treatment of uncomplicated Plasmodium falciparum malaria in two multi-country randomised clinical trials in African adults
Source: Malar J. 2014 Nov 25;13:458. doi: 10.1186/1475-2875-13-458 (PMC4364337; doi:10.1186/1475-2875-13-458)
Supplement: Supplementary file 1 — Additional file 1: Table S1: Median change from Baseline to last observation in laboratory values in study 1134. Description: The data in the table provides details of laboratory values from study 1134. (DOCX 38 KB) [file 12936_2014_3681_MOESM1_ESM.docx]

**Efficacy and safety of a combination of azithromycin and chloroquine for the treatment of uncomplicated *Plasmodium falciparum* malaria in two multi-country randomized clinical trials in African adults**

**Additional Table 1 Median change from baseline to last observation in laboratory values in study 1134**

|  | **AZCQ 1,000 mg** | | | **MQ 1,250 mg** | | |
| --- | --- | --- | --- | --- | --- | --- |
|  | **N** | **Baseline median** | **Median change from baseline** | **N** | **Baseline median** | **Median change from baseline** |
| Hemoglobin, g/dL | 96 | 13.2 | –0.4 | 99 | 13.1 | –0.2 |
| Hematocrit, % | 104 | 40.2 | –1.5 | 103 | 39.9 | –0.3 |
| Platelets, 10^3^/mm^3^ | 81 | 138 | 19 | 78 | 146 | 18 |
| WBC count, 10^3^/mm^3^ | 81 | 2.1 | –0.3 | 78 | 1.4 | 0.6 |
| Lymphocytes, absolute count, 10^3^/mm^3^ | 25 | 0.96 | 0.47 | 23 | 0.93 | 0.81 |
| Total neutrophils, absolute count, 10^3^/mm^3^ | 25 | 3.09 | –0.4 | 23 | 3.06 | –0.46 |
| Basophils, absolute count, 10^3^/mm^3^ | 5 | 1.9 | –0.9 | 4 | 0.74 | 0.01 |
| Eosinophils, absolute count, 10^3^/mm^3^ | 5 | 1.2 | 0.04 | 4 | 0.66 | 0.17 |
| Monocytes, absolute count, 10^3^/mm^3^ | 5 | 9.26 | –0.44 | 4 | 5.98 | 0.06 |
| Bilirubin, mg/dL |  |  |  |  |  |  |
| Total | 98 | 1.1 | –0.4 | 99 | 1.1 | –0.2 |
| Direct | 62 | 0.3 | –0.1 | 62 | 0.3 | 0 |
| AST (SGOT), IU/L | 88 | 40 | 1 | 89 | 39 | 1 |
| ALT (SGPT), IU/L | 107 | 29 | 5 | 107 | 25 | 0 |
| Alkaline phosphatase, IU/L | 32 | 34 | –3 | 35 | 34 | 0 |
| BUN, mg/dL | 37 | 28.3 | –0.4 | 44 | 28.6 | 0 |
| Creatinine, mg/dL | 48 | 1.2 | 0 | 49 | 1.2 | 0 |
| Sodium, mEq/L | 94 | 137 | 1 | 93 | 137 | 0 |
| Potassium, mEq/L | 93 | 4 | 0.3 | 93 | 4.1 | –0.1 |
| Chloride, mEq/L | 23 | 105 | 1 | 23 | 105 | 2 |
| Glucose (random), mg/dL | 73 | 87 | 1 | 71 | 84 | 5 |

ALT = alanine aminotransferase; AST = aspartate aminotransferase; AZCQ 1,000 mg = azithromycin 1,000 mg plus chloroquine 600-mg base; BUN = blood urea nitrogen; MQ = mefloquine hydrochloride; SGOT = serum glutamic oxaloacetic transaminase; SGPT = serum glutamic pyruvic transaminase; WBC = white blood cell.
