# Supplementary material for: Efficacy and safety of a combination of azithromycin and chloroquine for the treatment of uncomplicated Plasmodium falciparum malaria in two multi-country randomised clinical trials in African adults
Source: Malar J. 2014 Nov 25;13:458. doi: 10.1186/1475-2875-13-458 (PMC4364337; doi:10.1186/1475-2875-13-458)
Supplement: Supplementary file 2 — Additional file 2: Table S2: Median change from Baseline to last observation in laboratory values in study 1155. Description: The data in the table provides details of laboratory values from study 1155. (DOCX 35 KB) [file 12936_2014_3681_MOESM2_ESM.docx]

**Efficacy and safety of a combination of azithromycin and chloroquine for the treatment of uncomplicated *Plasmodium falciparum* malaria in two multi-country randomized clinical trials in African adults**

**Additional Table 2 Median change from baseline to last observation in laboratory values in study 1155**

|  | **AZCQ 1,000 mg** | | | **MQ 1,250 mg** | | |
| --- | --- | --- | --- | --- | --- | --- |
|  | **N** | **Baseline median** | **Median change from baseline** | **N** | **Baseline median** | **Median change from baseline** |
| Hemoglobin, g/dL | 108 | 14.9 | –0.2 | 109 | 14.7 | 0.0 |
| Hematocrit, % | 108 | 43.4 | –0.1 | 109 | 43.2 | 0.0 |
| RBC count | 108 | 4.96 | –0.03 | 109 | 4.98 | 0.03 |
| Platelets, 10^3^/mm^3^ | 107 | 185 | 13 | 108 | 172 | 17 |
| WBC count, 10^3^/mm^3^ | 108 | 5.7 | –0.4 | 109 | 5.0 | 0.2 |
| Lymphocytes, absolute count, 10^3^/mm^3^ | 63 | 1.45 | 0.40 | 62 | 1.30 | 0.51 |
| Total neutrophils, absolute count, 10^3^/mm^3^ | 61 | 3.95 | –0.49 | 58 | 3.75 | –0.37 |
| Basophils, % | 48 | 0.0 | 0.0 | 52 | 0.0 | 0.0 |
| Eosinophils, % | 48 | –0.2 | 0.9 | 52 | 0.6 | 0.9 |
| Monocytes, absolute count, 10^3^/mm^3^ | 55 | 0.56 | 0.09 | 53 | 0.46 | 0.09 |
| Bilirubin total, mg/dL | 108 | 1.0 | –0.3 | 109 | 0.8 | –0.2 |
| AST (SGOT), IU/L | 94 | 42 | –1 | 91 | 40 | –4 |
| ALT (SGPT), IU/L | 108 | 32 | 3 | 109 | 29 | –1 |
| Creatinine, mg/dL | 108 | 1.4 | 0.1 | 109 | 1.4 | 0.0 |

ALT = alanine aminotransferase; AST = aspartate aminotransferase; AZCQ 1,000 mg = azithromycin 1,000 mg plus chloroquine 600-mg base; MQ = mefloquine hydrochloride; RBC = red blood cell; SGOT = serum glutamic oxaloacetic transaminase; SGPT = serum glutamic pyruvic transaminase; WBC = white blood cell.
